# Transoral Laser Microsurgery for Early Glottic Cancer

**DOI:** 10.5402/2011/750676

**Published:** 2011-11-23

**Authors:** Lisha McClelland, Esmond Carr, Tawakir Kamani, Jennifer Cade, Kate Young, Sean Mortimore

**Affiliations:** Royal Derby Hospital, Uttoxeter Road, Derby DE22 3NE, UK

## Abstract

*Objectives.* To assess the outcome of transoral laser-assisted microsurgery (TLM) with regards to local and distant tumour control, quality of voice and swallowing. *Design.* Retrospective review of patients with five-year follow-up period. *Setting.* Royal Derby Hospital Head and Neck Department. *Participants.* All patients undergoing TLM with a diagnosis of Tis, T1, or T2 glottic tumour following endoscopic biopsy. *Main Outcome Measures.* Speech, swallowing, cancer-free survival, laryngectomy-free survival, and mortality rate. *Results.* 22 patients were treated for early glottic carcinoma with TLM. The 5-year local control rate for T1 tumours is 89% and 56% for T2 tumours. The laryngectomy rate was 4.5%. The mortality rate from local and distant disease was 4.5% with an overall mortality rate of 22% from all causes. 40% of patients had normal voices and a further 45% had only mild or moderate voice change. At their last followup, no patients assessed had any difficulty swallowing relating to their treatment for glottic cancer. *Conclusion.* Transoral Endoscopic CO2 laser microsurgery is a valid technique for treating early glottic tumours.

## 1. Introduction

Treatment of carcinoma in situ and early invasive squamous cell carcinoma of the larynx has changed significantly with the advent of transoral endoscopic CO_2_ laser microsurgery [1]. Historically early glottic tumours have been treated with radiotherapy [2]. It has proven efficacy and a perception of functionally better outcome, as no tissue is removed [3]. Transoral laser microsurgery of the larynx has been considered experimental and is not standard practice throughout UK. It has been used to debulk the larynx but with little confidence in the ability to completely resect tumours. The advantages to the patient include a short stay procedure, with little pain, avoidance of radiotherapy side effects, and voice preservation [4]. A short-stay procedure may have financial benefit for the hospital and patient compared with an average six-week course of radiotherapy which requires daily commute for outpatient treatment or an inpatient stay [2]. 

We present our experience of transoral laser microsurgery for glottic squamous cell carcinoma with regards to local and distant tumour control, quality of voice, and swallowing. 

## 2. Methods

### 2.1. Ethical Considerations

 There were no exclusion criteria for this study. All patients were offered treatment with a curative intent. All patients had full disclosure of available treatment options and counselling as to the advantages and disadvantages of each. The only potential problems we considered were the patient's general fitness to undergo anaesthesia and TLM or a radical course of radiotherapy. All our patients were considered suitable for either treatment. As both treatments were already established within the service, ethics approval was not required. Written informed consent was obtained from all patients. 

A retrospective study of all patients undergoing transoral laser microsurgery under the care of the senior author (S. Mortimore) with a diagnosis of Tis, T1, or T2 glottic tumour (TNM 6th Edition) from January, 2002 to December, 2004. Information was collected from our ENT laser database, oncology and pathology databases, and patient notes. Quality of voice was analysed by our speech and language therapist using the Oates Russell Voice Profile (The appendix), an overall grade system with scores of 1 to 5 [5]. Swallowing was also assessed using a standard bedside dysphagia assessment and cervical auscultation of swallow sounds. F. E. E. S (fiberoptic endoscopic evaluation of swallowing) was carried out to assess and manage dysphagia symptoms in most cases. Patients with T1 and T2 disease had radiological imaging of larynx, neck, and chest. Data was collected up to April, 2010.

## 3. Results

22 patients had TLM for early laryngeal cancer with an average age of 67 (Range 54–83). All patients had an initial endoscopy and either laser or conventional biopsy to confirm the diagnosis. The T staging for the early glottis tumours staged 4 patients with Tis, 9 patients with T1 disease (T1a = 6, T1b = 3), and 9 patients with T2 tumours. No patients had clinical or radiological evidence of nodal disease or distant metastasis. The average number of laser procedures was 1.87 (Range 1–5). All patients either drank alcohol or smoked tobacco. 16 patients were treated effectively with TLM alone. 6 patients underwent further treatment with TLM for residual disease following their initial treatment. Five patients had treatment for recurrent disease, 3 with TLM, 1 with radiotherapy, and 1 with both TLM and chemoradiotherapy. 1 patient had recurrent disease which was not resectable endoscopically. This patient had a total laryngectomy and bilateral selective neck dissection followed by radiotherapy. Unfortunately, they were unable to complete the radiotherapy course, as they were medically unfit and later died with residual disease. One patient had a metachronous tongue tumour and one a synchronous lung tumour which later metastasised. Two patients with T2 disease developed nodal disease and underwent bilateral selective neck dissections. 

16 patients were surgically and pathologically clear of tumour. 6 had dysplasia at resection margins and underwent second look procedures and 4 with further resection. 

Voice outcomes were assessed with the Oates Russell Voice Profile, 8 patients had normal voices, and 7 had mild changes. 2 had moderate and 3 moderate-to-severe voice changes. There were no aphonic patients. 1 patient died of other causes prior to having voice analysis by a speech and language therapist (SLT), and 1 had a laryngectomy. The voice outcome roughly correlates with T staging (Table 1). Swallowing assessment was predominantly postoperative; however, a few patients with preoperative dysphagia were seen before surgery. First assessment was in nearly all cases 1-2 days postoperatively. In cases where the patient was unwell the first assessment was up to one week after surgery. Patients whose resection only involved a small part of the vocal folds recovered normal eating and drinking 1–3 weeks after surgery. Patients with larger resections generally required 2-3 months to reach their potential. Patients with large resections required a longer period of swallowing rehabilitation to learn and review compensation strategies. A few patients required up to 8–10 contacts over approx 6 months to reach their potential. At their last followup, 19 patients had returned to a normal diet with no discernable difficulty swallowing. One patient had previous treatment for a metachronous base of tongue tumour resulting in swallowing difficulty and was fed via gastrostomy tube. Two patients died of unrelated disease before swallowing was fully assessed. 5 patients died within the 5-year follow-up period, and only one death was due to disease recurrence. The remaining patients are all disease free. The 5-year local control rate for T1 tumours is 89% and 56% for T2 tumours. The laryngeal preservation rate is 100% for T1 and 89% for T2 tumours. The mortality rate from local or distant disease is 4.5% with an overall disease-free survival rate of 77% at 5 years. 

## 4. Discussion

Transoral laser microsurgery can be used in any patient who is fit for general anaesthesia. The only major technical difficulty can come with anterior commissure tumours. Posttreatment surveillance may be easier following TLM, as the oedema and mucosities associated with EBRT are absent [2]. Submucosal disease may be difficult to detect with either modality. 

TLM and EBRT offer comparable cure rates for early glottic tumours [6]. Comparing the treatment modalities is problematic. TLM allows for accurate staging of the tumour (during resection), whilst radiotherapy relies on the initial microlaryngoscopy for clinical staging. With transoral laser microsurgery, the tumour is resected until the tissue feels and appears healthy under the microscope. A second-look procedure is common if there is any concern over histological margins, residual or recurrent disease, or failure to improve postoperatively. The ability to perform repeat laser resections should be viewed as a benefit of surgery rather than a failure of the procedure. 

In traditional resection methods, the excision line represents the extent of resection, and any histological evidence of tumour at the margins means that the tumour has not been adequately resected. With laser resection, there is a zone of thermal injury to either side of the excision line, and it may, therefore, be difficult for a histopathologist to say with certainty that the tumour is fully excised. It is also difficult in the larynx to give the usually accepted millimetres of clearance [7]. Frozen section has in the past been advocated; however, we feel it does not add to the intraoperative management. It may be helpful to send separate specimens from the edge of the resected area which is macroscopically clear of tumour [8]. Transoral laser microsurgery, therefore, relies heavily on the surgeon making a competent assessment of the resection. 

There is no universally agreed time frame for which disease is counted as residual disease as opposed to recurrence. Residual disease suggests that the tumour was incompletely excised; perhaps the size was underestimated or the tumour understaged. Recurrence conversely means that the tumour was completely excised previously. We have used a 1-year cutoff point with tumour recurring in the first twelve months classified as residual disease and tumour after this time being recurrence. 

There is no universally accepted scoring system for voice analysis. Our speech and language therapists have previously used generic voice scoring systems such as GRBAS but have found them inflexible and, therefore, have adopted the Oates Russell Voice Profile. Subjective voice rating is performed by experienced voice therapists. The grading score of 0 to 5 correlates clinically with a normal voice to severe dysphonia [5]. Patients were given postoperative voice care involving routine hydration and humidification (humidified air) whilst on the ward. At home, patients were given clear instructions regarding steam inhalation 3-4 times per day, conservative voice use in the first week, and routine voice care advice. Voice therapy was carried out with patients as required. Communication needs were monitored and strategies offered as required such as amplifiers or amplified telephones for patients with permanently compromised vocal loudness. Patients were seen within the MDT review or directly by the SLT and clinical nurse specialist who conduct the laser follow-up clinic in our service so long-term needs were addressed as they arose. The quality of voice after laser resection reflects the degree of resection required with more superficial tumours having a better outcome [9, 10]. Interestingly, there was no significant variation if the anterior commissure was involved although this may reflect the small number of cases in this group [11]. Voice outcome is not routinely assessed following radiotherapy in our hospital. The literature suggests that voice outcomes in TLM may be either comparable or better compared with EBRT [5, 6]. 

Swallowing outcomes following TLM are good and recovery time relatively short for early glottic tumours compared to the prolonged discomfort of mucosities and dryness or altered taste following EBRT, which may persist indefinitely [12]. 

The literature suggests a five-year survival after EBRT to be 85% for T1-T2 glottic tumours, and the rate with TLM is comparable [13]. Total laryngectomy rates are quoted as 4–6% after open surgery and 9–12% after radiotherapy [13, 14]. We have only looked at early glottic tumours so a lower laryngectomy rate would be expected as is the case.

## 5. Conclusion

Transoral endoscopic CO_2_ laser microsurgery is a valid technique for treating early glottic tumours. It has good outcomes for local and distant disease control [15]. TLM can be used to treat residual, recurrent, or new tumours even in a previously treated radiotherapy fields. Speech and swallowing results are comparable or better than EBRT.

## Figures and Tables

**Table 1:** T stage and voice outcome.

T stage	Voice outcome							
	0	1	2	3	4	5	Valve	U/A
Tis	1	1	1	1	0	0	0	0
T1	5	2	1	0	0	0	0	1
T2	2	0	2	1	3	0	1	0

## Appendix

### Oates Russell Voice Profile

(0) Normal,
(1) mild change-v. occ heard/harsh,
(2) mild/mod, intermittent, harsh/diplophonic,
(3) moderate-persistent, harsh,
(4) mod/severe-persistent, severe whispery,
(5) severe-aphonic.
